# Exercise and eating behaviors among Austrian transgender and gender-diverse adolescents

**DOI:** 10.1007/s00431-025-06014-x

**Published:** 2025-02-13

**Authors:** Sarah Knaus, Friedrich Teutsch, Jo Steininger, Diana Esteve Alguacil, Stefan Riedl

**Affiliations:** 1 Department of Pediatrics and Adolescent Medicine, Medical University of Vienna, Währinger Gürtel 18-20, 1090 Vienna, Austria; 2 Austrian National Public Health Institute (Gesundheit Österreich GmbH), Vienna, Austria; 3 University of Valencia, Valencia, Spain; 4 St. Anna Children’s Hospital, Vienna, Austria

**Keywords:** Adolescence, Exercise, Obesity, Gender diversity, Social inclusion, Gendered embodiment

## Abstract

Transgender and gender-diverse (TGD) adolescent populations are at a higher risk for obesity. The aim of this study was to explore possible reasons, such as differences in eating and exercise behaviors. This was a prospective cross-sectional study of TGD adolescent patients at the pediatric endocrinology outpatient clinic of the Vienna General Hospital from January to July 2022. Patients were included in the World Health Organization’s Health Behaviour in School-Aged Children (HBSC) survey, which assesses for multiple measures including exercise, eating, and mental wellbeing. We recruited 32 patients via the outpatient clinic, and a further 55 adolescents self-identified as TGD through the survey. Responses from the cohort of 87 TGD adolescents were compared to the Austrian sample containing 10,110 participants. TGD adolescents reported significantly lower levels of physical activity than the national sample, both in instances of vigorous activity (*p* = 0.002) and any physical activity lasting > 60 min (*p* < 0.001) per week. Gender variance was here demonstrated to be a strong predictor for physical inactivity levels, even when correcting for low mental health scores. Regarding body image, TGD participants were also more likely to describe themselves as feeling “too fat” (*p* = 0.001). No statistically significant difference in eating behavior was found.

*Conclusion*: The results of this study point towards the complexity of healthcare needs in the TGD adolescent population. The interconnectedness of mental health and exercise behavior is well described. However, qualitative work is needed to understand the specific relationship between gender expression, body image, eating and exercise behaviors, as well as social inclusion.

**Table:** 

**What is Known:** *• Transgender and gender-diverse (TGD) adolescents are at an increased risk for obesity. The causes for this are most likely complex, and remain poorly understood.* *• Survey studies in the United States and New Zealand have reported reduced levels of physical activity in the TGD population. However, data is scarce, particularly regarding adolescents*.	
**What is New:** *• This prospective, national survey study is the first to demonstrate reduced levels of self-reported physical activity in a cohort of central European TGD adolescents*.

**What is Known:**

*• Transgender and gender-diverse (TGD) adolescents are at an increased risk for obesity. The causes for this are most likely complex, and remain poorly understood.*

*• Survey studies in the United States and New Zealand have reported reduced levels of physical activity in the TGD population. However, data is scarce, particularly regarding adolescents*.

**What is New:**

*• This prospective, national survey study is the first to demonstrate reduced levels of self-reported physical activity in a cohort of central European TGD adolescents*.

## Introduction

As a gender minority, transgender and gender-diverse (TGD) people experience significantly worse health outcomes across many different measures [1, 2]. This has often been theorized within the minority stress model, which describes how the cumulative effect of structural inequality and social discrimination that gender minorities face over the course of their lifetime results in barriers to healthcare, decreased mental health, and, on average, lower socioeconomic status [3–6]. In particular, auxological data has shown gender variance to be a risk factor for obesity — independent of psychiatric morbidity, and from a young age [7–10]. The reasons for this are not entirely clear, and most likely complex [11].

Experiencing obesity or overweight has far-reaching health implications for TGD adolescents. Firstly, the consequences of weight-based stigmatization in healthcare must be accounted for [12, 13]. Obesity can affect how and whether TGD individuals receive gender-affirming care. Though long-term evidence is still scarce, treatment protocols with gender-affirming steroid hormone therapy (GAHT) [1] have documented metabolic and cardiovascular side effects, among them is an increase in body weight and visceral body fat [14–18]. Access to gender-affirming surgery is further commonly subject to body mass index (BMI) cutoffs, citing technical limitations and higher rates of complications [19–21], often without a clear evidence base [22]. Additionally, obesity can have a significant impact on TGD adolescents’ psychosocial development and mental health, as TGD students are at an increased risk for being bullied due to weight or size compared to their cisgender classmates [23]. Taken together, these findings can be understood through an intersectional theoretical lens, as being overweight can place gender and sexual minorities in an additionally marginalized position [24, 25].

Research exploring the potential drivers of overweight in TGD adolescents, or rather differences in body weight and frame more generally, is scarce. Some evidence points to behavioral differences in gender minority youth, such as a higher prevalence of unhealthy weight-regulating behaviors [8], as well as a lower likelihood to regularly engage in strenuous exercise [7, 26]. Qualitative studies and case reports [9, 11] as well as one large online survey [27] have also discussed a possible correlation between gender dysphoria and both restrictive and binge-eating disorders, suggesting that the profound body image dissatisfaction many TGD adolescents experience could put them at risk for pathological eating behavior. On a structural level, the practice of splitting group sports and school physical education by sex/gender continues to systematically discriminate against, and subsequently exclude, TGD adolescents, limiting their access to regular exercise [28].

Most studies on the topic of gender diversity and body weight have so far been conducted in the USA, and to our knowledge, there is currently no data on central European TGD youth. The objective of this cross-sectional, observational study was therefore to screen Austrian TGD adolescents for behavioral differences commonly associated with effects on body weight and frame: physical activity levels and nutritional intake [29]. To account for the complex, bidirectional associations between weight-regulating behaviors, mental illness, and socioeconomic status, we also considered general mental health, experiences of bullying, body image, and family affluence in our calculations [30, 31]. We hypothesized that TGD adolescents would report lower overall levels of physical activity than the national sample, as well as higher levels of nutritional intake. We posited that these differences would be significant even when correcting for signs of poor mental health. We intend for the results of this study to increase understanding of the specific needs of the TGD adolescent population, encouraging healthcare practitioners to screen for unhealthy weight-regulating behaviors and provide exercise opportunities for young people seeking gender-affirming care.

## Methods

### Survey tool

This study was conducted as a collaboration with the Health Behaviour in School-Aged Children (HBSC) survey. First initiated by the World Health Organization (WHO) in 1983, the HBSC survey is an international research study aiming to increase understanding of health behaviors, health, well-being, lifestyles, and social contexts of young people across different countries. The HBSC survey is conducted simultaneously in 50 countries across Europe and North America at 4-year-intervals and includes upwards of 300,000 children and adolescents. Austria has taken part in the HBSC project since its initiation. Execution is funded by the Austrian Federal Ministry for Social Affairs, Health, Care, and Consumer Protection and coordinated by the Austrian National Public Health Institute (Gesundheit Österreich GmbH). The latest sample was recruited over the school year 2021/2022 [32–34].

The HBSC survey broadly examines eating behaviors, obesity, physical activity and sedentary behavior, body image, weight reduction behavior, oral health, bullying and fighting, self-rated health, life satisfaction, socioeconomic environment, substance abuse, sexual behavior, school environment, and relationships with family and peers. For this particular study, our main aim was to compare TGD adolescents and the national sample on measures relating weight regulation, which is why we focused on nutrition and eating behaviors (self-reported consumption of fast food, fruits, vegetables, sweets, energy drinks, and sugared soft drinks), exercise behavior (self-reported vigorous activity and instances of physical activity > 60 min per week), as well as body image. To account for the complexity of healthcare needs and social vulnerability in the TGD adolescent cohort, we also examined aggregated scores regarding mental health (self-reported life satisfaction, the WHO-5 Well-Being Index, screening questions for depressive symptoms, feelings of social exclusion and loneliness) as well as experiences of bullying (instances of being victim and/or perpetrator of bullying, either in-person or online). Other factors assessed by the survey were not analyzed for this study.

Most of the questions factored into this analysis were posed in a way which prompted participants to quantify certain behaviors or feelings within a given time frame. For assessing exercise and eating habits, this entailed counting instances of certain types of food consumption or exercise per week. To screen for mental wellbeing, the survey requires participants to report whether symptoms and feelings such as poor sleep, irritability, or loneliness were experienced “never–rarely–less/more than half of the time–mostly–always” over the past 6–12 months. The question on body image required participants to state whether they regarded their bodies as “(much) too thin,” “just right,” or “(much) too fat.”

The Austrian HBSC survey, as of 2021/2022, asks participants about their gender identity (German “*Geschlecht*”) without explicitly differentiating between sex and gender, providing the following answers: male, female, and “other (if so, specify).” The third option provides a possibility to investigate TGD adolescents separately; however, as is the case with all self-report nationwide surveys using one-step measures of sex and gender, it runs the risk of missing a population of binary transgender adolescents who identify simply as male or female.

For surveying adolescent health and health behavior in Austria, the HBSC questionnaire is adapted to three different groups: middle school students (5th and 7th grade), high school students (9th and 11th grade), as well as working young adults (German “*Lehrberufe*”). In our cohort of TGD adolescents, we used the form for 7th grade for ages 13–14 years, 9th grade for ages 15–17 years, and 11th grade for ages 17–18 years in adolescents attending school. Working young adults received the form for working young adults (“*Lehrberufe*”).

### Participants

This study was designed as an addendum to the larger HBSC data collection. To ensure a solid sample to compare with the representational national sample of Austrian youth (recruited via schools across the country), a proportion of TGD patients was recruited separately through the outpatient clinic for Pediatric Endocrinology at the University Clinic for Child and Adolescent Health in Vienna. Our transgender sample was registered with a separate identification number, to ensure that gender minorities were not epidemiologically overrepresented in the national sample. Participants were instructed to answer the question regarding their gender identity (“*Geschlecht*”) as “other” and then specify either trans man or trans woman.

All TGD adolescents aged 13–18 years presenting to the outpatient clinic for from January 2022 to July 2022 were asked to take part in this study. Inclusion criteria were (a) presentation to clinic for assessment regarding gender incongruence or gender dysphoria and (b) age 13–18 years. Exclusion criteria were (a) chromosomal or other genetic variants, (b) typical or atypical (late-onset) congenital adrenal hyperplasia, and (c) other differences in sex development (DSD).

We decided to forgo a psychiatrically confirmed diagnosis of ‘transsexualism’ (International Classification of Diseases 10th Revision; ICD-10) as prerequisite for inclusion into this study, since this diagnosis is usually made after a lengthy, multidisciplinary process in order to ensure diagnostic certainty before commencing GAHT. Adolescents who seek care for gender incongruence represent a gender minority regardless of whether they eventually qualify for hormone treatment.

Participation was strictly voluntary, and informed consent to publish was attained both from the participants and their legal guardians. The study was approved by the ethics committee of the Medical University of Vienna (ECS 2231/2021) in accordance with the Declaration of Helsinki.

### Data protection

HBSC survey data is recorded anonymously via an online questionnaire. The database is overseen by a Database Manager. A pseudonymous record of participants was kept locally.

### Data analysis

Statistical software used for analysis included Excel, SPSS, and Jamovi. Chi-square and T-tests were conducted to compare the TGD collective with the national sample. Linear regression modeling was used to examine the significance of gender variance as an independent variable opposite mental health. To account for multiple testing, all calculations were subject to a Bonferroni correction, with statistical significance thus defined as *p* < 0.003.

## Results

### Sample characteristics

The Austrian HBSC questionnaire was used to survey a total of 10,110 participants in 2021/2022. Of the 87 adolescents classified as TGD, 32 had been recruited separately through the outpatient clinic, and a further 55 were identified via their answers on the questionnaire. Average age of the total Austrian sample was 15.6 years (standard deviation 2.73) and 16.1 years (standard deviation 1.93) for the TGD cohort. Aggregated family affluence scores did not differ significantly between the TGD and national sample, though there was a trend towards slightly lower self-reported family affluence in the TGD cohort (Pearson’s Chi-square value 7.633, *p* = 0.022).

### Nutrition

The HBSC survey approximates healthy eating behavior by asking participants to report consumption frequency of the following food categories over the course of a week: fast food, fruits, vegetables, sweets, sugared soft drinks, and energy drinks. In this analysis, no difference was found between the TGD cohort and the national sample on self-reported consumption of fast food (Pearson’s Chi-square value 4.063, *p* = 0.13), fruits (1.334, *p* = 0.51), vegetables (0.497, *p* = 0.78), sweets (3.596, *p* = 0.17), and sugared soft drinks (5.314, *p* = 0.07). There was a trend towards higher self-reported consumption of energy drinks in the TGD cohort, though after Bonferroni correction, this result was not statistically significant (7.827, *p* = 0.02).

### Physical activity

**Fig. 1:**
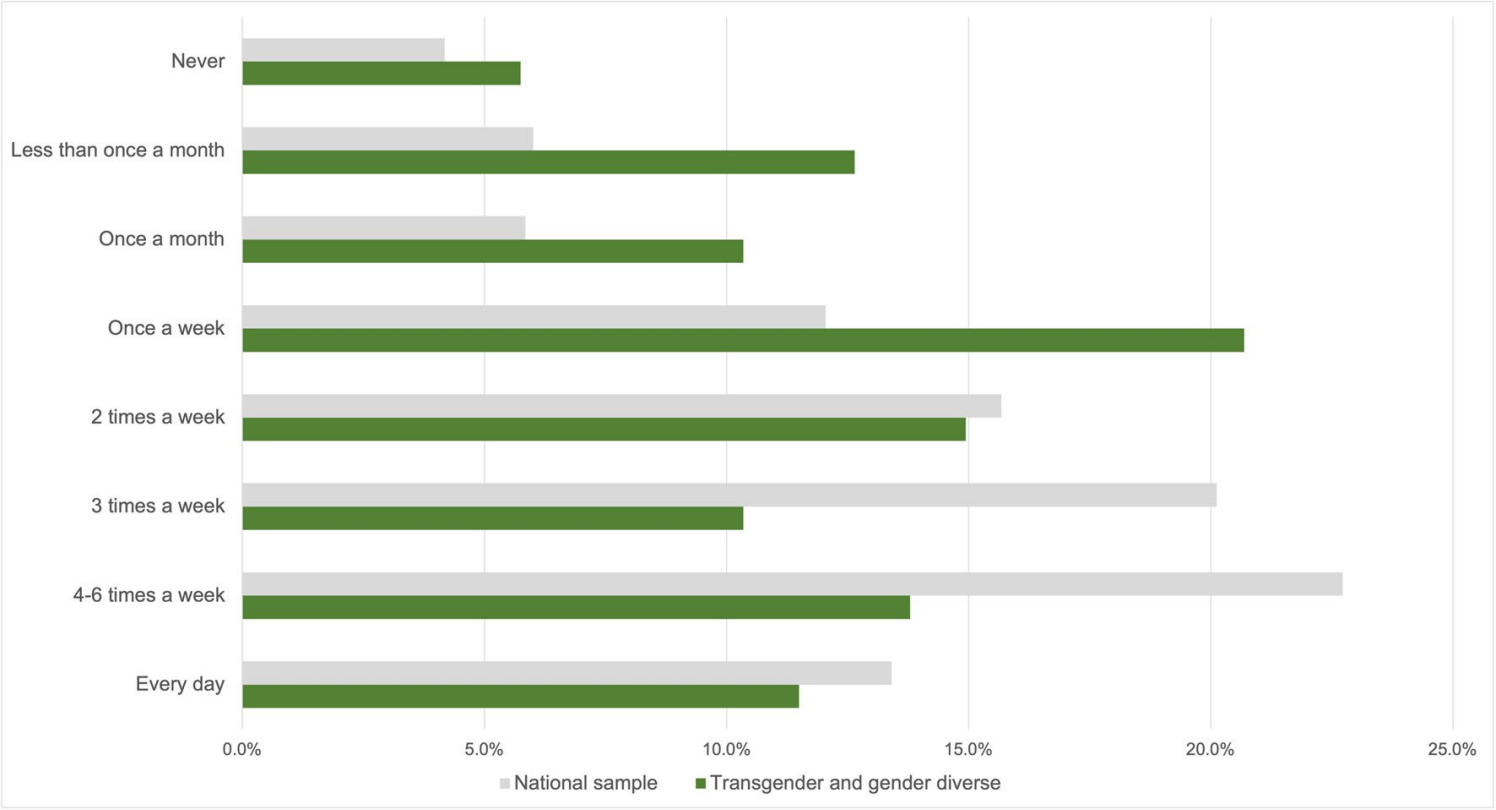
Instances of vigorous physical activity: graph comparing national sample and transgender and gender-diverse participants’ responses to the question “In your free time, how often are you normally physically active to the point of exertion (sweating, out of breath)?”

**Fig. 2:**
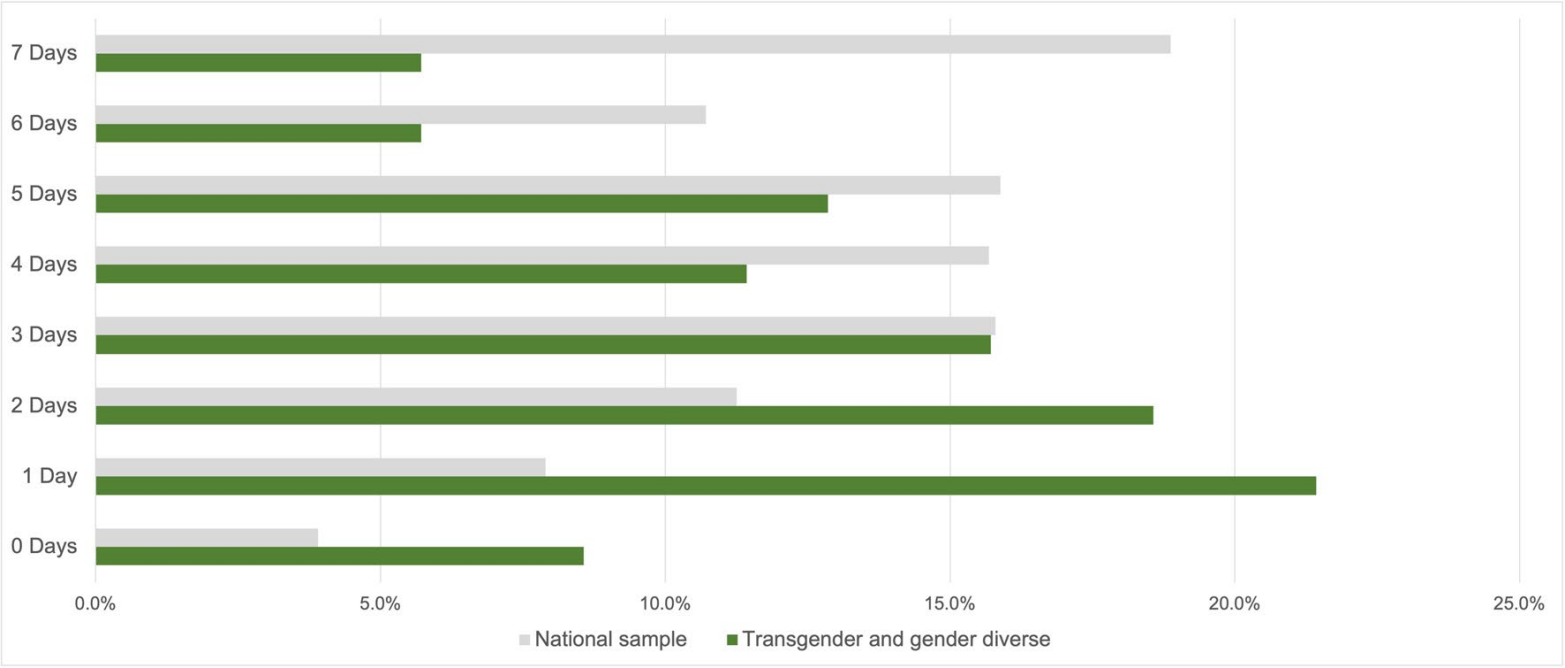
Comparison between national sample and transgender and gender-diverse adolescents’ responses to the question “On how many days in the past week were you physically active for at least 60 min?”

Levels of physical activity are assessed by asking participants to estimate how often they normally engage in vigorous physical activity in an average month, as well to quantify instances any kind of physical activity lasting 60 min over the past 7 days. Complete data was available for a total of 9993 adolescents. Within the TGD cohort specifically, it was not possible to calculate the association between BMI and self-reported physical activity levels, due to the fact that weight status is normed for sex assigned at birth, which was an unknown variable in the TGD sample. Across the national sample, however, lower self-reported levels of physical activity were significantly associated with higher self-reported BMI (Pearson’s Chi-square value 53.2, *p* < 0.001 and 54.4 < *p* < 0.001, respectively), demonstrating a connection between physical inactivity and overweight. Chi-square tests comparing the TGD subgroup to the national sample meanwhile revealed significant differences, with TGD participants reporting fewer instances of both vigorous activity (22.474, *p* = 0.002; see Fig. 1) as well as instances of any physical activity lasting at least 60 min over the course of a week (32.116, *p* < 0.001; see Fig. 2).

### Psychological wellbeing, body image, and bullying

A large portion of the HBSC survey is dedicated to mental health. Screening questions include a general question about life satisfaction on a 0–10 scale, the WHO-5 Well-Being Index [35] (including self-reported instances of feeling “cheerful,” “calm and relaxed,” “active and vigorous”), as well as screening questions for depressive symptoms (instances of “feeling low,” irritability, difficulties sleeping, anxiety) and loneliness.

TGD adolescents reported significantly lower rates of overall life satisfaction (*p* < 0.001), were more likely to show signs of depression via the WHO-5 Well-Being Index (*p* < 0.001), and reported more feelings of social exclusion and loneliness over the past 12 months (Pearson’s chi-square value 47.287, *p* < 0.001). In an aggregated mental health score, binarized to “high/low,” 65.9% of TGD youth scored “low,” as opposed to 23.2% in the national sample (85.277, *p* < 0.001).

Linear regression modeling was applied to account for the effects of aggregated mental health scores, life satisfaction, gender variance, and body image on physical activity. Here, gender variance proved to be a strong risk factor regarding lower self-reported instances of vigorous physical activity (see Table 1), while regarding daily instances of physical activity > 60 min, mental health scores proved to be a stronger factor (see Table 2).

**Table 1:** Linear regression model coefficient: vigorous physical activity (*N* = 9478/*R*^2^ = 0.12)

Predictor	Estimate	SE	*t*	*p*	Stand. Estimate
Intercept	5.3186	0.07200	73.87	< 0.001	
Mental health score (WHO5)	− 0.0194	0.00100	− 19.28	< 0.001	− 0.2315
Life satisfaction	− 0.0568	0.01159	− 4.90	< 0.001	− 0.0578
TGD (1–0)	− 0.6167	0.03773	− 16.35	< 0.001	− 0.3233
Body image (1–0)	− 0.1270	0.03806	− 3.34	< 0.001	− 0.0666

**Table 2:** Linear regression model coefficient: daily physical activity > 60 min (*N* = 6630/*R*^2^ = 0.142)

Predictor	Estimate	SE	*t*	*p*	Stand. Estimate
Intercept	1.9842	0.09588	20.70	< 0.001	
Mental health score (WHO5)	0.0258	0.00131	19.74	< 0.001	0.2847
Life satisfaction	0.0591	0.01533	3.86	< 0.001	0.0544
TGD (1–0)	0.4291	0.04838	8.87	< 0.001	0.2090
Body image (1–0)	0.2541	0.04837	5.25	< 0.001	0.1238

The HBSC questionnaire features one question specifically on body image, asking participants to rate whether they feel “much/a bit too thin,” “just right,” or “much/a little too fat.” Here, 15.3% of TGD adolescents stated they felt “too thin,” 27.1% felt “just right,” and 57.6% felt “too fat.” This contrasted with the national sample insofar as the proportion of adolescents who considered themselves “too fat” was significantly larger (Pearson’s chi-square value 15.049, *p* = 0.001; see Table 3).

**Table 3:** Comparison of self-reported body image between transgender and gender-diverse adolescents and the national sample

	Transgender and gender diverse	National sample
“(Much) too thin”	15.3%	18.9%
“Just right”	27.1%	43.6%
“(Much) too fat”	57.6%	37.4%

In our linear regression model, body image was binarized by grouping those who considered their body ‘normal’ vis-á-vis those who did, body image satisfaction proved to have an effect on self-reported daily levels of physical activity > 60 min (see Table 2) but less so on instances of vigorous activity (see Table 1).

Experiences of bullying were assessed by asking participants to report whether they had had been victims or perpetrators of bullying in the past months. Results were grouped into “yes,” defined as 2–3 × /month or more, or “rarely/no,” defined as 1–2 × /month or less. There were no statistically significant differences between the TGD group and the national sample in self-reported frequency of having been bullied or bullying others at school (Pearson’s Chi-square value 1.218, *p* = 0.270 and 2.776, *p* = 0.096, respectively). TGD adolescents reported significantly higher levels of having experienced cyber-bulling (16.055, *p* < 0.001), but no increased tendency to engage in cyber-bullying themselves (0.005, *p* = 0.944).

## Discussion

Previous auxological studies on TGD adolescent populations have shown discrepancies in BMI between TGD adolescents and their cisgender peers [36]. Some of this data also suggests that being part of a gender minority could be an independent risk factor for obesity, even when accounting for the elevated levels of psychiatric morbidity in the TGD population [10]. The reasons are largely unclear. This study offers a partial explanation, by demonstrating significantly lower levels of self-reported physical activity in TGD adolescent populations when compared to a representative national sample. These findings are consistent with survey studies conducted in the USA [26, 27] and are, to our knowledge, the first from central Europe. We found no statistically significant differences in self-reported eating behaviors between the two groups, though considering that disordered eating has been described in TGD adolescent populations [27, 37], we would caution that our relatively small sample size may not have been able to adequately reflect this aspect.

A further finding in this study, though not the primary object of investigation, was significantly worse self-reported mental health across every measure. TGD adolescents also scored significantly worse on self-rated body image. This data is unfortunately not surprising, as it is consistent with a plethora of existing research surrounding gender variance and psychiatric morbidity in both adults and adolescents [5, 14]. Linear regression modeling demonstrated that gender variance remained a significant predictor of lower levels of vigorous physical activity when controlling for aggregated mental health scores.

### Limitations

This study has several limitations. Firstly, as a self-report, multiple choice survey, it can only ever approximate behaviors and never fully represent the lived experiences of participants. Secondly, in the national sample, due to the phrasing of the question assessing for gender identity (“*Geschlecht*”), it runs the risk of overlooking a significant number of TGD adolescents in the population at large. Given that survey studies and census data from the UK, Canada, New Zealand, and the USA estimate the proportion of gender diversity to be between 0.5 and 2% of the young population [38–42], with the Austrian HBSC survey yielding a self-reported percentage of 0.5%, this could likely be the case. Finally, as mentioned above, due to the relatively small number of TGD participants in total, the small sample size may have also under-powered us for demonstrating statistically significant differences in eating and drinking behaviors, such as the tendency towards higher energy drink consumption in the TGD subgroup.

### Implications

The implications of our findings are complex. To preface, we have no way of extrapolating the reasons why Austrian TGD adolescents engage in less exercise than cisgender youth from this data. On the most basic level, we believe it could point to the ways in which splitting sports by sex/gender from school age to competitive levels continues to exclude some TGD individuals from physical education [28].

Secondly, taken together with the striking differences in self-reported mental health and body image, trans adolescents’ exercise habits may also reflect the complex, bidirectional relationships between psychological distress and social participation more broadly [43]. Generally speaking, individuals affected by mental illness are two to three times more likely to be overweight [44] and engage in less regular exercise [45]. Conversely, people with obesity are up to 70% more at risk to suffer from mental illness [46].

The need for mental health support in the chronically underserved TGD community is apparent and has been discussed. It is, however, also important to stress that wellbeing does not exist in a vacuum. Gender diversity per se is not a mental illness, and the distress felt by many TGD individuals must be understood within the context of social marginalization [47]. Overall, given the well-documented positive relationship between exercise and mental health, which has also been demonstrated for adult gay, lesbian, and bisexual people [48], we propose that TGD adolescents could benefit both physically and psychosocially from group exercise programs tailored to their needs.

Finally, there is still very little work within the medical field on the particular relationship between body weight, frame, and gender identity [49]. It is necessary here to gesture towards the troubled history of medical transgender research, as a field which has been largely disavowed by transgender scholarship “for its stigmatizing and pathologizing construction of transness, for its sometimes implicit and often explicit racism, for its relationship to colonialism and global flows of capital, and for other related reasons.” [50, p. 6]. Despite a recent paradigm shift in the empirical sciences away from “gender deviance” towards “gender difference,” the pathologizing lens of medical transgender research lives on in the form of its “damage-centered” approach, which tends to be overly focused on documenting the effects of adversities and suffering associated with being an oppressed minority [51]. This form of research has alienated large portions of the TGD community, ultimately hampering the development of more interdisciplinary concepts of gender in healthcare [50]. Consequently, the relationship between gender dysphoria, body image, and weight-regulating behaviors remains poorly understood within the medical scientific community [11, 52].

Studying weight and weight-related behaviors of TGD adolescents taps into a complex, sensitive, and oftentimes highly personal discourse surrounding gendered embodiment — meaning the multitude of ways that the body comes into contact with and is shaped by its environment, including cultural experiences [53]. We argue that the field of medical transgender research must develop a non-pathologizing understanding of the significance of body weight for gender expression and gendered embodiment. This would encompass an attempt to learn about and understand the highly individual, non-medical methods of body modification that TGD people employ to achieve increased levels of gender congruence without pathologizing them [54]. More broadly, it would also entail thinking about overweight and obesity beyond notions of ‘health vs illness’. Survey studies such as this one, while useful for quantifying group differences and comparing them to national averages, are inadequate at capturing the more nuanced conversation surrounding gender, body image, and eating and exercise behaviors. TGD people are rightfully wary of this form of research [50, 55]. We would like to encourage the medical community to embrace more mixed methods approaches to transgender research, including qualitative research, in order to do justice to the complexity of the topic.

## Conclusion

This study provides the first data on eating and exercise behaviors in central European TGD adolescents. Our findings are in line with other surveys reporting reduced levels of physical activity of TGD adolescents, highlighting the complexity of healthcare needs in this underserved and vulnerable community. Above all, we encourage an ethics of care that keeps in mind to what end and for whom research is conducted. We hope that the results of this study might aid practitioners in providing more comprehensive and inclusive care for TGD adolescents and act as a starting point for developing more in-depth work on gendered embodiment.

## Data Availability

The data that support the findings of this study are available upon request under 10.5281/zenodo.13341995.

## Abbreviations

BMI: Body mass index
GAHT: Gender-affirming hormone therapy
HBSC: Health Behaviour in School-Aged Children
ICD-10: International Classification of Diseases, 10th Revision
TGD: Transgender and gender diverse
WHO: World Health Organization

## References

[1] Coleman E, Radix AE, Bouman WP, Brown GR, de Vries ALC, Deutsch MB, Ettner R et al (2022) Standards of care for the health of transgender and gender diverse people, version 8. Int J Transgend Health 23:S1-s259. 10.1080/26895269.2022.2100644 36238954 10.1080/26895269.2022.2100644 PMC9553112

[2] Safer JD, Tangpricha V (2019) Care of transgender persons. N Engl J Med 381:2451–2460. 10.1056/NEJMcp1903650 31851801 10.1056/NEJMcp1903650

[3] Meyer IH (2003) Prejudice, social stress, and mental health in lesbian, gay, and bisexual populations: conceptual issues and research evidence. Psychol Bull 129:674–697. 10.1037/0033-2909.129.5.674 12956539 10.1037/0033-2909.129.5.674 PMC2072932

[4] Jaffee KD, Shires DA, Stroumsa D (2016) Discrimination and delayed health care among transgender women and men: implications for improving medical education and health care delivery. Med Care 54:1010–1016. 10.1097/mlr.0000000000000583 27314263 10.1097/MLR.0000000000000583

[5] Safer JD, Coleman E, Feldman J, Garofalo R, Hembree W, Radix A, Sevelius J (2016) Barriers to healthcare for transgender individuals. Curr Opin Endocrinol Diabetes Obes 23:168–171. 10.1097/med.0000000000000227 26910276 10.1097/MED.0000000000000227 PMC4802845

[6] Reisner SL, Poteat T, Keatley J, Cabral M, Mothopeng T, Dunham E, Holland CE, Max R, Baral SD (2016) Global health burden and needs of transgender populations: a review. Lancet 388:412–436. 10.1016/s0140-6736(16)00684-x 27323919 10.1016/S0140-6736(16)00684-X PMC7035595

[7] Lucassen MF, Guntupalli AM, Clark T, Fenaughty J, Denny S, Fleming T, Smith M, Utter J (2019) Body size and weight, and the nutrition and activity behaviours of sexual and gender minority youth: findings and implications from New Zealand. Public Health Nutr 22:2346–2356. 10.1017/s1368980019001149 31159912 10.1017/S1368980019001149 PMC10260432

[8] Guss CE, Williams DN, Reisner SL, Austin SB, Katz-Wise SL (2017) Disordered weight management behaviors, nonprescription steroid use, and weight perception in transgender youth. J Adolesc Health 60:17–22. 10.1016/j.jadohealth.2016.08.027 28029539 10.1016/j.jadohealth.2016.08.027 PMC8091135

[9] Avila JT, Golden NH, Aye T (2019) Eating Disorder Screening in Transgender Youth. J Adolesc Health 65:815–817. 10.1016/j.jadohealth.2019.06.011 31500946 10.1016/j.jadohealth.2019.06.011

[10] Knaus S, Steininger J, Klinger D, Riedl S (2024) Body mass index distributions and obesity prevalence in a transgender youth cohort - a retrospective analysis. J Adolesc Health 75:127–132. 10.1016/j.jadohealth.2024.03.005 38752964 10.1016/j.jadohealth.2024.03.005

[11] Williams DR, Chaves E, Greenwood NE, Kushner J, Chelvakumar G, Swaringen SE, Leibowitz SF (2022) Care of gender diverse youth with obesity. Curr Obes Rep 11:215–226. 10.1007/s13679-022-00480-2 36050541 10.1007/s13679-022-00480-2

[12] Lee KM, Hunger JM, Tomiyama AJ (2021) Weight stigma and health behaviors: evidence from the Eating in America Study. Int J Obes 45:1499–1509. 10.1038/s41366-021-00814-5 10.1038/s41366-021-00814-5 PMC8236399 33934109

[13] Paine EA (2021) “Fat broken arm syndrome”: negotiating risk, stigma, and weight bias in LGBTQ healthcare. Soc Sci Med 270:113609. 10.1016/j.socscimed.2020.113609 33401217 10.1016/j.socscimed.2020.113609 PMC7920326

[14] de Souza SR, Frank AP, Nelson MD, Garcia MM, Palmer BF, Clegg DJ (2017) Sex, gender, and transgender: metabolic impact of cross hormone therapy. Adv Exp Med Biol 1043:611–627. 10.1007/978-3-319-70178-3_27 29224113 10.1007/978-3-319-70178-3_27

[15] Streed CG Jr, Beach LB, Caceres BA, Dowshen NL, Moreau KL, Mukherjee M, Poteat T, Radix A, Reisner SL, Singh V (2021) Assessing and addressing cardiovascular health in people who are transgender and gender diverse: a scientific statement from the American Heart Association. Circulation 144:e136–e148. 10.1161/cir.0000000000001003 34235936 10.1161/CIR.0000000000001003 PMC8638087

[16] Defreyne J, Van de Bruaene LDL, Rietzschel E, Van Schuylenbergh J, T’Sjoen GGR (2019) Effects of gender-affirming hormones on lipid, metabolic, and cardiac surrogate blood markers in transgender persons. Clin Chem 65:119–134. 10.1373/clinchem.2018.288241 30602477 10.1373/clinchem.2018.288241

[17] Suppakitjanusant P, Ji Y, Stevenson MO, Chantrapanichkul P, Sineath RC, Goodman M, Alvarez JA, Tangpricha V (2020) Effects of gender affirming hormone therapy on body mass index in transgender individuals: a longitudinal cohort study. J Clin Transl Endocrinol 21:100230. 10.1016/j.jcte.2020.100230 32685379 10.1016/j.jcte.2020.100230 PMC7358708

[18] Kyinn M, Banks K, Leemaqz SY, Sarkodie E, Goldstein D, Irwig MS (2021) Weight gain and obesity rates in transgender and gender-diverse adults before and during hormone therapy. Int J Obes (Lond) 45:2562–2569. 10.1038/s41366-021-00935-x 34400797 10.1038/s41366-021-00935-x

[19] Linsenmeyer WR, Garwood S (2023) Patient-centered approaches to using BMI to evaluate gender-affirming surgery eligibility. AMA J Ethics 25:E398-406. 10.1001/amajethics.2023.398 37285293 10.1001/amajethics.2023.398

[20] Martinson TG, Ramachandran S, Lindner R, Reisman T, Safer JD (2020) High body mass index is a significant barrier to gender-confirmation surgery for transgender and gender-nonbinary individuals. Endocr Pract 26:6–15. 10.4158/ep-2019-0345 31461357 10.4158/EP-2019-0345

[21] Taormina JM, Iwamoto SJ (2023) Filling a gap in care: addressing obesity in transgender and gender diverse patients. Int J Obes (Lond) 47:761–763. 10.1038/s41366-023-01334-0 37414875 10.1038/s41366-023-01334-0 PMC11210399

[22] Brownstone LM, DeRieux J, Kelly DA, Sumlin LJ, Gaudiani JL (2021) Body mass index requirements for gender-affirming surgeries are not empirically based. Transgend Health 6:121–124. 10.1089/trgh.2020.0068 34414267 10.1089/trgh.2020.0068 PMC8363993

[23] Puhl RM, Himmelstein MS, Watson RJ (2019) Weight-based victimization among sexual and gender minority adolescents: implications for substance use and mental health. Health Psychol 38:727–737. 10.1037/hea0000758 31157534 10.1037/hea0000758 PMC7194133

[24] Lawrence SE, Watson RJ, Eadeh HM, Brown C, Puhl RM, Eisenberg ME (2024) Bias-based bullying, self-esteem, queer identity pride, and disordered eating behaviors among sexually and gender diverse adolescents. Int J Eat Disord 57:303–315. 10.1002/eat.24092 37990394 10.1002/eat.24092 PMC10922269

[25] White F (2019) Embodying the fat/trans intersection. In: Friedman MCR, Rinaldi J (eds) Thickening fat: fat bodies, intersectionality, and social justice, 1st ed. Routledge, London, pp 110–121

[26] Bishop A, Overcash F, McGuire J, Reicks M (2020) Diet and physical activity behaviors among adolescent transgender students: school survey results. J Adolesc Health 66:484–490. 10.1016/j.jadohealth.2019.10.026 31959401 10.1016/j.jadohealth.2019.10.026

[27] Roberts SR, Salk RH, Thoma BC, Romito M, Levine MD, Choukas-Bradley S (2021) Disparities in disordered eating between gender minority and cisgender adolescents. Int J Eat Disord 54:1135–1146. 10.1002/eat.23494 33638569 10.1002/eat.23494 PMC13051521

[28] Jones BA, Arcelus J, Bouman WP, Haycraft E (2017) Sport and transgender people: a systematic review of the literature relating to sport participation and competitive sport policies. Sports Med 47:701–716. 10.1007/s40279-016-0621-y 27699698 10.1007/s40279-016-0621-y PMC5357259

[29] Weinsier RL, Hunter GR, Heini AF, Goran MI, Sell SM (1998) The etiology of obesity: relative contribution of metabolic factors, diet, and physical activity. Am J Med 105:145–150. 10.1016/s0002-9343(98)00190-9 9727822 10.1016/s0002-9343(98)00190-9

[30] Anekwe CV, Jarrell AR, Townsend MJ, Gaudier GI, Hiserodt JM, Stanford FC (2020) Socioeconomics Obesity Curr Obes Rep 9:272–279. 10.1007/s13679-020-00398-7 32627133 10.1007/s13679-020-00398-7 PMC7484407

[31] Avila C, Holloway AC, Hahn MK, Morrison KM, Restivo M, Anglin R, Taylor VH (2015) An overview of links between obesity and mental health. Curr Obes Rep 4:303–310. 10.1007/s13679-015-0164-9 26627487 10.1007/s13679-015-0164-9

[32] Teutsch F, Felder-Puig R, Winkler R (2022) Dokumentation der GÖG-Erhebungen 2021/22 zur Jugendgesundheit (HBSC und Lehrlinge). Bundesministerium für Soziales, Gesundheit, Pflege und Konsumentenschutz, Vienna

[33] Felder-Puig R, Teutsch F, Winkler R (2023) Gesundheit und Gesundheitsverhalten von österreichischen Lehrlingen. Ergebnisse der Lehrlingsgesundheitsbefragung 2021/22. Bundesministerium für Soziales, Gesundheit, Pflege und Konsumentenschutz, Vienna. https://goeg.at/sites/goeg.at/files/Österr.%20Lehrlingsgesundheitsbericht%202023.pdf. Accessed 5 Feb 2025

[34] Felder-Puig R, Teutsch F, Winkler R (2023) Gesundheit und Gesundheitsverhalten von österreichischen Schülerinnen und Schülern. Ergebnisse des WHO-HBSC-Survey 2021/22. Bundesministerium für Soziales, Gesundheit, Pflege und Konsumentenschutz, Vienna. https://goeg.at/sites/goeg.at/files/inline-files/Österr._HBSC-Bericht_2022.pdf. Accessed 5 Feb 2025

[35] Topp CW, Østergaard SD, Søndergaard S, Bech P (2015) The WHO-5 Well-Being Index: a systematic review of the literature. Psychother Psychosom 84:167–176. 10.1159/000376585 25831962 10.1159/000376585

[36] Fornander MJ, Roberts T, Egan AM, Moser CN (2022) Weight status, medication use, and recreational activities of treatment-naïve transgender youth. Child Obes 18:228–236. 10.1089/chi.2021.0155 34762510 10.1089/chi.2021.0155

[37] Pham AH, Eadeh HM, Garrison MM, Ahrens KR (2023) A longitudinal study on disordered eating in transgender and nonbinary adolescents. Acad Pediatr 23:1247–1251. 10.1016/j.acap.2022.12.013 36587733 10.1016/j.acap.2022.12.013

[38] Shields JP, Cohen R, Glassman JR, Whitaker K, Franks H, Bertolini I (2013) Estimating population size and demographic characteristics of lesbian, gay, bisexual, and transgender youth in middle school. J Adolesc Health 52:248–250. 10.1016/j.jadohealth.2012.06.016 23332492 10.1016/j.jadohealth.2012.06.016

[39] Clark TC, Lucassen MF, Bullen P, Denny SJ, Fleming TM, Robinson EM, Rossen FV (2014) The health and well-being of transgender high school students: results from the New Zealand adolescent health survey (Youth’12). J Adolesc Health 55:93–99. 10.1016/j.jadohealth.2013.11.008 24438852 10.1016/j.jadohealth.2013.11.008

[40] Statistics Canada (2021) Understanding who we are: sex at birth and gender of people in Canada. 2021 Census, Ottawa, Canada. https://www12.statcan.gc.ca/census-recensement/2021/ref/98-500/014/98-500-x2021014-eng.cfm. Accessed 5 Feb 2025

[41] Herman J, Flores, AR, Brown, TN, Wilson, BD, & Conron, KJ (2017) Age of individuals who identify as transgender in the United States. UCLA: the Williams Institute, Los Angeles, CA. https://williamsinstitute.law.ucla.edu/publications/trans-adults-united-states. Accessed 5 Feb 2025

[42] Kidd KM, Sequeira GM, Douglas C, Paglisotti T, Inwards-Breland DJ, Miller E, Coulter RWS (2021) Prevalence of gender-diverse youth in an urban school district. Pediatrics 147:1–3. 10.1542/peds.2020-049823 10.1542/peds.2020-049823 PMC8168604 34006616

[43] Cameron AJ, Magliano DJ, Dunstan DW, Zimmet PZ, Hesketh K, Peeters A, Shaw JE (2012) A bi-directional relationship between obesity and health-related quality of life: evidence from the longitudinal AusDiab study. Int J Obes (Lond) 36:295–303. 10.1038/ijo.2011.103 21556045 10.1038/ijo.2011.103

[44] De Hert EM, Correll CU, Bobes J, Cetkovich-Bakmas M, Cohen D, Asai I, Detraux J, Gautam S, Möller HJ, Ndetei DM, Newcomer JW, Uwakwe R, Leucht S (2011) Physical illness in patients with severe mental disorders. I. Prevalence, impact of medications and disparities in health care. World Psychiatry 10:52–77. 10.1002/j.2051-5545.2011.tb00014.x 21379357 10.1002/j.2051-5545.2011.tb00014.x PMC3048500

[45] Vancampfort D, Firth J, Schuch FB, Rosenbaum S, Mugisha J, Hallgren M, Probst M, Ward PB, Gaughran F, De Hert M, Carvalho AF, Stubbs B (2017) Sedentary behavior and physical activity levels in people with schizophrenia, bipolar disorder and major depressive disorder: a global systematic review and meta-analysis. World Psychiatry 16:308–315. 10.1002/wps.20458 28941119 10.1002/wps.20458 PMC5608847

[46] Rindler GA, Gries A, Freidl W (2023) Associations between overweight, obesity, and mental health: a retrospective study among European adults aged 50. Front Public Health 11 10.3389/fpubh.2023.1206283 10.3389/fpubh.2023.1206283 PMC10390701 37533526

[47] Winter S, Diamond M, Green J, Karasic D, Reed T, Whittle S, Wylie K (2016) Transgender people: health at the margins of society. Lancet 388:390–400. 10.1016/s0140-6736(16)00683-8 27323925 10.1016/S0140-6736(16)00683-8

[48] Pharr JR, Flatt JD, Chien LC, Kachen A, Olakunde BO (2021) Exercise as a mitigator of poor mental health among lesbian, gay, and bisexual adults. J Phys Act Health 18:548–556. 10.1123/jpah.2020-0703 33848980 10.1123/jpah.2020-0703

[49] Pham A, Kerman HM, Albertson K, Crouch JM, Inwards-Breland DJ, Ahrens KR (2022) Understanding the complex relationship between one’s body, eating, exercise, and gender-affirming medical care among transgender and nonbinary adolescents and young adults. Transgend Health 8(2):149–158. 10.1089/trgh.2021.0112 10.1089/trgh.2021.0112 PMC10066775 37013089

[50] Billard T, Everhart A, Zhang E (2022) Whither trans studies? On fields, post-disciplines, and the need for an applied transgender studies. 1:1–18. 10.57814/pe84-4348

[51] Riggs DW, Rosenberg S (2020) Trans young people and embodiment. In: Wyn J, Cahill H, Cuervo H (eds) Handbook of children and youth studies, 2nd ed. Springer Nature Singapore, Singapore, pp 1–12

[52] Tabaac A, Perrin PB, Benotsch EG (2018) Discrimination, mental health, and body image among transgender and gender-non-binary individuals: constructing a multiple mediational path model. J Gay Lesbian Soc Serv 30:1–16. 10.1080/10538720.2017.1408514 30880881 10.1080/10538720.2017.1408514 PMC6417421

[53] Gattario KH, Frisén A, Teall TL, Piran N (2020) Embodiment: cultural and gender differences and associations with life satisfaction. Body Image 35:1–10. 10.1016/j.bodyim.2020.07.005 32877841 10.1016/j.bodyim.2020.07.005

[54] Griffiths S, Yager Z (2019) Gender, embodiment, and eating disorders. J Adolesc Health 64:425–426. 10.1016/j.jadohealth.2019.01.016 30904090 10.1016/j.jadohealth.2019.01.016

[55] Minalga B, Chung C, Davids JD, Martin A, Perry NL, Shook A (2022) Research on transgender people must benefit transgender people. Lancet 399:628. 10.1016/s0140-6736(21)02806-3 35151395 10.1016/S0140-6736(21)02806-3

